# PDMS–Epoxy Micro-Nano Composite Structures Constructed via Open-Loop Addition Reactions and Their Optical and Antifouling Performance Modulation

**DOI:** 10.3390/ma19061244

**Published:** 2026-03-21

**Authors:** Chao Xu, Xiaofan Chen, Shimin Zhai, Dan Wang, Ruofei Zhu

**Affiliations:** 1 Xinjiang Key Laboratory of Intelligent and Green Textile, College of Textile & Clothing, Xinjiang University, Urumqi 830017, China; 2 College of Textile Science and Engineering, Zhejiang University of Technology, Hangzhou 310000, China; 3 State Key Laboratory of Molecular Engineering of Polymers, Fudan University, Shanghai 200433, China

**Keywords:** epoxy resin, PDMS, ring-opening reaction, functionalized composite coating, easy-to-clean

## Abstract

Epoxy resin (E-51) exhibits excellent adhesion and is widely used in the preparation of functional composite coatings. However, its smooth surface lacking micro/nano composite structures limits its self-cleaning capability and optical properties. Direct incorporation of organic silicone or inorganic fillers often faces severe phase separation and filler agglomeration issues, resulting in defects in coating durability and weather resistance. To address these challenges, this study developed a synergistic modification strategy integrating surface energy modulation with the architectural design of micro/nano-structures. Amino-terminated PDMS undergoes ring-opening addition reactions with epoxy groups in the epoxy resin, while functionalized barium sulfate nanoparticles modified with dual silane coupling agents are incorporated to enhance optical properties. This synergistic approach not only resolved interfacial compatibility but also endowed the PDMS@EP-BaSO_4_ coating with outstanding comprehensive properties; the water contact angle increased to 123.5°, demonstrating an easy-to-clean benefit. Visible light reflectance reached 95%, and emissivity rose to 90%. Furthermore, when applied to metal surfaces, the coating exhibited excellent stability against acid–alkali–salt corrosion, extreme temperatures, and ultrasonic agitation. This work provided a novel approach for developing protective coatings that integrated high reflectance, high emissivity, and long-term anti-soiling properties.

## 1. Introduction

Metal materials form the foundation of modern industry, yet they face the dual challenge of resource scarcity and service-induced degradation. During prolonged use, metals are frequently exposed to complex environments, confronting issues such as atmospheric corrosion, UV aging, stress fatigue from thermal cycling, and contaminant adhesion. Notably, coating materials have emerged as a key solution to mitigate outdoor environmental impacts on metals due to their ease of adhesion to substrates, low cost, convenient application, and broad substrate compatibility [1,2,3,4]. Recent years have seen the development of multifunctional coating materials, including metallic and alloy coatings [5], organic coatings [6], ceramic and hard coatings [7,8,9], and composite coatings [10]. Although various coatings have been developed to enhance the outdoor service performance of metallic materials, issues such as contaminant adhesion and stress fatigue caused by high and low temperatures remain major concerns reported in most studies, significantly reducing the service life of metallic materials. This impact is particularly pronounced in regions with large diurnal temperature variations and high levels of dust and sand.

To tackle the challenges mentioned above, a novel strategy for constructing multifunctional composite coatings has been proposed [11]. By incorporating targeted functional materials such as inorganic nanoparticles and organosilicon polymers into conventional coatings, the effects of extreme temperatures are mitigated while enhancing self-cleaning capabilities to prevent contaminant adhesion. For instance, Li et al. [12] prepared a composite graphene/epoxy radiation heat dissipation coating with high emissivity and strong adhesion, significantly increasing the substrate’s emissivity to 0.93. As demonstrated by Wang et al. [13], by modifying the chemical functional groups and surface morphology of PDMS-modified MPC coatings, MPC was successfully transformed from hydrophilic to highly hydrophobic. However, PDMS and epoxy resin exhibit significant differences in solubility. Physical blending between the two readily leads to macroscopic phase separation, causing coating defects and even substantial degradation in mechanical properties, thereby failing to meet environmental requirements in practical applications. Moreover, inadequate compatibility between inorganic nanoparticles and organic matrices in most composite coatings leads to filler agglomeration and insufficient dispersion within the system. This results in diminished performance and limits practical application scenarios. Among established composite coating materials, epoxy resin is widely adopted as a substrate due to its superior adhesion, excellent chemical resistance, and favorable mechanical properties. However, most epoxy resin coatings exhibit poor toughness and inadequate compatibility with functional fillers, hindering their sustained protective efficacy [14]. Therefore, there is a need to develop a composite coating material featuring outstanding compatibility, superior photothermal regulation capabilities, strong self-cleaning properties, and excellent mechanical performance.

In light of the aforementioned issues, this work proposes a strategy based on ring-opening addition reactions and the construction of micro-nano composite structures to engineer epoxy resins into composite coatings with enhanced protective properties. By chemically modifying epoxy resin with amino-terminated PDMS and dual silane-coupled functionalized barium sulphate, organic silicone segments and inorganic nanoparticles are chemically bonded into the epoxy matrix. This approach resolved phase separation issues arising from physical blending of PDMS and epoxy resin, enabling uniform dispersion of inorganic nanoparticles within the matrix and establishing a foundation for metal protection. Furthermore, the PDMS@EP-BaSO_4_ coating exhibits exceptionally high reflectance and emissivity within the visible spectrum, highlighting its highly efficient passive cooling capability. Notably, this dense functional composite coating maintained ultra-high reflectance exceeding 90% on average, even after 24 h of exposure to strong acids, strong alkalis, high-salinity environments, high-temperature heating, or low-temperature freezing. Multi-scenario simulation experiments further validated the stability of the composite coating in addressing outdoor metal protection. Interestingly, through synergistic interaction between the dense coating and inorganic nanoparticles, the PDMS@EP-BaSO_4_ coating exhibited self-cleaning properties. This work offers novel insights for developing metal protective coatings, with these multifunctional applications expected to meet the long-term protective requirements for metals in outdoor environments.

## 2. Experimental Section

### 2.1. Materials

Epoxy resin (E-51), isophorone diamine,methyl orange (MO), (Shanghai McLean Biochemical Technology Co, Ltd., Shanghai, China), amine-propyl double-terminated polydimethylsiloxane (Mn ≈ 2500), KH-560, KH-550 (Nanjing Shuguang Chemical Group Co., Ltd., Nanjing, China), barium sulfate, anhydrous ethanol, copper sulphate, and cobalt nitrate (Tianjin Zhiyuan Chemical Co., Ltd., Tianjin, China) were used.

### 2.2. Preparation of Epoxy-Modified Barium Sulfate

Barium sulfate was pretreated by drying and grinding. Then, in an acidic (pH 3.0–3.5) alcohol–water system (alcohol:water = 9:1, ethanol 9 mL, water 1 mL), KH-550 was hydrolyzed at 1% by weight of barium sulfate for 30–60 min. Subsequently, 1 g of the pretreated barium sulfate was added to the hydrolysate and stirred at a constant temperature of 50–60 °C in a water bath for 2–3 h. The aqueous solution of the KH-550-modified barium sulfate intermediate was centrifuged at 4000 r/min for 10 min, washed three times with anhydrous ethanol, vacuum-dried at 80 °C for 6–8 h, and ground to obtain the modified barium sulfate intermediate powder. Subsequently, KH-560 was hydrolyzed under identical conditions. The modified barium sulfate intermediate from KH-550 was added to this hydrolysate and stirred at a constant temperature of 55–65 °C in a water bath for 2.5–3.5 h. Finally, the post-reaction solution underwent centrifugation at 4000 r/min for 10 min, was washed three times with anhydrous ethanol, and was vacuum-dried at 80 °C for 6–8 h to yield the modified BaSO_4_.

### 2.3. Preparation of PDMS@EP-BaSO_4_ Composite Coating

Disperse 0.2 g of epoxy-modified BaSO_4_ nanoparticles in 3 mL of anhydrous ethanol, add a small amount of silane coupling agent KH-560, and sonicate for 30 min to obtain a uniform BaSO_4_ dispersion. Dissolve 4.5 g of epoxy resin E-51 in 2 mL of anhydrous ethanol and stir magnetically for 10 min at 50 °C in a water bath. Next, slowly add 1.5 g of amino-terminated polydimethylsiloxane (Amino-PDMS) to the resin solution while gradually raising the temperature to 60 °C. React for 1 h to promote the ring-opening addition reaction between the amino and epoxy groups. After the reaction, the BaSO_4_ nanoparticles dispersion was poured into the modified resin system. Stirring was continued for 20 min at constant temperature to ensure uniform dispersion of the filler. Once the mixture cooled to room temperature, 1 g of isophorone diamine was added and stirred vigorously for 5 min to achieve thorough mixing. The prepared adhesive solution was degassed in a vacuum oven for 10 min and then applied to the pretreated substrate surface using a squeegee coating method. Finally, the coating was cured using a stepwise heating program: first held at 40 °C for 1 h to remove solvents and then heated to 50 °C for 2 h of curing. After natural cooling to room temperature, the PDMS@EP-BaSO_4_ composite coating was obtained.

### 2.4. Testing and Characterization

The microstructure of the sample surface was observed using a field emission scanning electron microscope (SEM, SU8010, Hitachi, Tokyo, Japan). Functional groups and chemical bonds in the samples were characterized using a Fourier transform infrared spectrometer (FTIR, VERTEX 70 RAMI, Bruker, Ettlingen, Germany). Elemental analysis of the samples was performed using X-ray photoelectron spectroscopy (XPS, ESCALAB 250Xi, Thermo, Waltham, MA, USA) with a metallographic microscope (M20-3M180, Shenzhen, China). Spectra were analyzed and fitted using Avantage 6.9.0 software. The wettability of the different coated samples was characterized at room temperature using a contact angle measuring instrument (JC2000DM, Shanghai Zhongchen Digital Technology Co., Ltd., Shanghai, China) with a 5 μL droplet. For each sample, at least three independent specimens were tested to ensure reproducibility.

The wavelength scan range of the ultraviolet–visible spectrophotometer (UV-Vis, TU-1901, Beijing Purkinje General Instrument Co., Ltd., Beijing, China) was set to 380–780 nm with a slow scan speed. Samples were cut into small circular pieces with a diameter of 1 cm and placed on the sample stage for testing. The average solar reflectance ρ_solar_ was obtained from Equation (1):

(1) $$\rho_{\mathrm{solar}}\;=\;\frac{\int_{\lambda 1}^{\lambda 2}\rho (\lambda )E(\lambda )d\lambda }{\int_{\lambda 1}^{\lambda 2}E(\lambda )d\lambda }$$

where ρ(λ): reflectance measured at a specific wavelength; E(λ): standard solar spectral irradiance (typically referenced from the ASTM G173 standard [15]); λ: wavelength range. The reflectance ε of the sample was measured using a diffuse reflectance cell of a Fourier transform infrared spectrometer, and the emissivity was calculated using the formula ε = 1 − ρ.

The mechanical durability of the PDMS@EP-BaSO_4_ composite coating was evaluated through systematic reciprocating abrasion testing. This procedure broadly followed the ASTM G99 [16] and ASTM D4060 [17] wear test methods, with adaptations made for the coating surface. To ensure repeatability, the coated surface was positioned face-down on P200-grade silicon carbide abrasive paper with an average particle size D50 = 75 ± 2 μm. A constant normal load of 5 N was uniformly applied across a 25 × 25 mm^2^ area of the specimen surface. The abrasion process employed a reciprocating cyclic mode, with each cycle comprising a 10 cm horizontal stroke followed by a 10 cm vertical stroke. The sliding speed was maintained at approximately 0.02 m per second. The WCA was measured after every 10 reciprocations, totaling 100 reciprocations, to assess the evolution of surface wettability under sustained mechanical stress. For scratch resistance testing, the coating surface was subjected to knife-scratch testing using a stainless steel blade. Each cycle involved 10 repeated scratches on the coating surface to simulate damage from sharp objects during actual use. The WCA of the coating was measured after each cycle for a total of 10 cycles. For the UV stability test, the coating was exposed to a UV lamp (1 W/cm^2^ and 340 nm wavelength) for 100 min, and the WCA was recorded every 10 min.

The self-cleaning capability of the coating surface was systematically assessed using multiple simulated contaminants, including inorganic salts (copper sulphate, cobalt nitrate), organic dyes (methyl orange), and natural soil. Samples were positioned at a fixed inclination angle of approximately 15°. At ambient temperature, pollutants of equal mass were distributed across the coating surface and allowed to settle for 10 min. Subsequently, a continuous rinse with deionized water at a flow rate of 2 mL/s was applied for 30 s.

Infrared thermography was employed to evaluate the influence of the PDMS@EP-BaSO_4_ composite coating on the thermal conductivity of metallic substrates. Metal specimens coated with the composite were placed upon a thermostatic heating platform maintained at 40 °C. An infrared thermal imaging camera continuously monitored and recorded the surface temperature variations in the specimens over the heating duration. Concurrently, uncoated bare iron plates served as control samples to assess the thermal response characteristics of the coatings.

The thermal stability of the coating was characterized using a thermogravimetric analyzer (HITACHI STA7300, Tokyo, Japan). Approximately 5 mg of the dried coating sample was placed within an alumina crucible. Under a nitrogen atmosphere (flow rate of 50 mL/min), the temperature was raised from room temperature to 800 °C at a heating rate of 20 °C/min.

To verify the adhesion strength of the coating, we employed the standard test methods for determining coating adhesion strength as per ASTM D4541 [18] and ASTM D3359 [19]. This involved repeatedly applying and removing adhesive tape from the coated surface to observe whether the coating detached with the tape. Contact angle measurements were taken every ten cycles to characterize the adhesion.

## 3. Results and Discussion

### 3.1. Structural and Morphological Analysis of PDMS@EP-BaSO_4_ Coating

**Figure 1:**
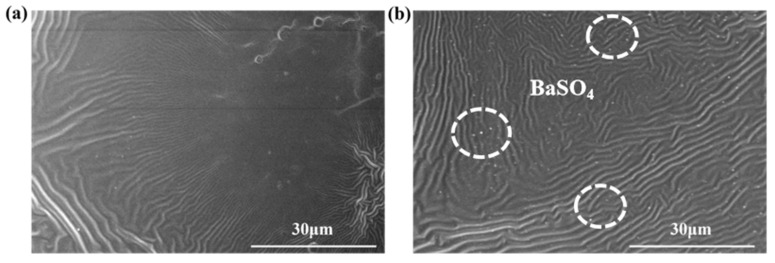
SEM images of (**a**) PDMS@EP and (**b**) PDMS@EP-BaSO_4_ coating.

Using SEM characterization of the coating surface morphology, the influence of introducing inorganic nanoparticle fillers on the microstructural evolution of the composite coating was revealed. Figure 1a shows that the PDMS@EP coating without BaSO_4_ nanoparticles exhibited a unique smooth, corrugated-like structure. This was primarily attributed to microphase separation between PDMS and EP during curing, coupled with the release of internal stresses caused by differences in thermal expansion coefficients and curing shrinkage rates between the components. Following the incorporation of epoxy-functionalized BaSO_4_ nano-inorganic particles, the coating surface morphology undergoes significant alteration. As illustrated in Figure 1b, the micro-nano filler particles dispersed uniformly and densely within the resin folds, forming a coarse topography with a multi-level micro-nano composite structure [20]. BaSO_4_ nanoparticles uniformly distributed across the 292–650 nm scale were observable on the coating surface. Moreover, the BaSO_4_ nanoparticles exhibited excellent interface bonding with the matrix, with no noticeable particle agglomeration or phase separation observed in the coating. This confirms that the silane coupling agent significantly enhanced the interfacial compatibility between the inorganic nanoparticles and the epoxy resin through chemical coupling. This micro-nano composite structure, synergistically constructed by chemically induced wrinkles and inorganic particles, provides the fundamental physical basis for the high contact angle and excellent self-cleaning performance of the coating [21].

**Figure 2:**
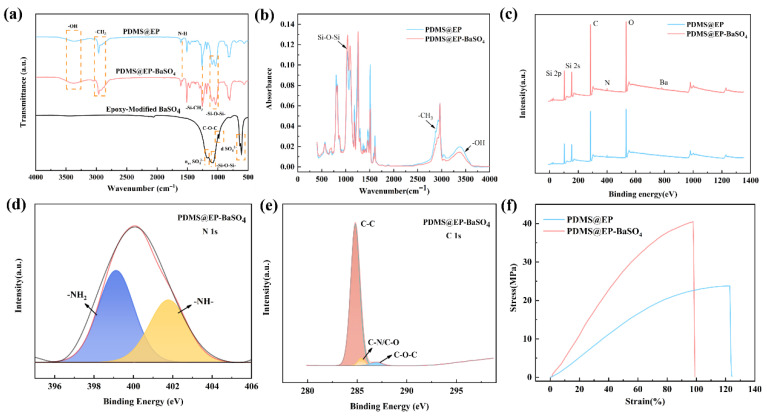
(**a**) FTIR spectra of PDMS@EP, PDMS@EP-BaSO_4_ and epoxy-modified BaSO_4_. (**b**) Quantitative FTIR absorption spectra of PDMS@EP and PDMS@EP-BaSO_4_. (**c**) Full XPS spectra of PDMS@EP and PDMS@EP-BaSO_4_ coating. (**d**) N 1s and (**e**) C 1s fine spectra of PDMS@EP-BaSO_4_. (**f**) Tensile stress–strain curves for PDMS@EP and PDMS@EP-BaSO_4_ coating.

The FTIR spectrum of epoxy-modified BaSO_4_ nanoparticles shown in Figure 2a clearly exhibited strong absorption bands for the asymmetric stretching vibrations of SO_4_^2−^ at 1075 cm^−1^ and 1175 cm^−1^, along with split bending vibration peaks at 610 cm^−1^ and 640 cm^−1^, all of which were consistent with BaSO_4_ inorganic nanoparticles. More notably, the modified spectrum exhibited characteristic signals from organic functional groups, including a weak C-O-C peak near 910 cm^−1^, confirming the successful incorporation of epoxy groups. Concurrently, the appearance of a stretching vibration near 1040 cm^−1^, characteristic of Si-O-Si bonds, indicates that the silane coupling agent had formed chemically bonded structures on the particle surface via a hydrolytic condensation reaction. Collectively, these analytical results confirm the successful grafting of epoxy groups onto the BaSO_4_ surface. The infrared spectra of PDMS@EP and PDMS@EP-BaSO_4_ both exhibit distinct C-H stretching vibration peaks (-CH_3_) at approximately 2960 cm^−1^, broad hydroxyl (-OH) peaks near 3400 cm^−1^, and -Si-CH_3_ peaks near 1260 cm^−1^. This provides preliminary confirmation of the epoxy resin–PDMS composite structure. Comparing the two spectra, the PDMS@EP-BaSO_4_ curve exhibited characteristic strong absorption bands in the 1000–1200 cm^−1^ range. Although the Si-O-Si bonds of PDMS were also absorbed in this region, the BaSO_4_-modified PDMS@EP-BaSO_4_ curve showed significantly broadened peak shapes in this interval. This broadening was attributed to the overlapping absorption of S-O bonds in the modified BaSO_4_, confirming the successful incorporation of inorganic nanoparticles [22]. Furthermore, the altered shape of the -OH peak at 3400 cm^−1^ indicated the presence of intermolecular hydrogen bonding interactions between the surface-modified groups of the inorganic nanoparticles and the resin matrix. Based on this, all spectra were normalized using the stable aromatic C=C stretching vibration peak at 1508 cm^−1^ as an internal standard (Figure 2b) [23]. Following the addition of BaSO_4_ nanoparticles, the relative intensity of the 1080 cm^−1^ peak corresponding to SO_4_^2−^ and Si–O–Si increased by 15.0%, confirming the successful incorporation of the filler. The relative intensities of -CH_3_ (2960 cm^−1^) and -OH (3400 cm^−1^) decreased significantly. This quantitative trend was attributed to the combined effects of volume exclusion and surface masking: as BaSO_4_ nanoparticles occupied part of the volume, the density of resin-derived organic functional groups decreased accordingly.

Figure 2c shows the full XPS spectrum scan; it clearly revealed characteristic peaks for Si, C, N, and O elements in the PDMS@EP sample. With the incorporation of modified BaSO_4_ nanoparticles, a characteristic Ba peak at 780 eV appears in the PDMS@EP-BaSO_4_ sample spectrum, confirming the presence of BaSO_4_ in the coating. To further elucidate the chemical crosslinking mechanism between E-51 epoxy resin and amino-terminated PDMS, high-resolution narrow-scan and peak-by-peak fitting analyses were performed on the N 1s and C 1s spectra of the PDMS@EP-BaSO_4_ sample. As shown in Figure 2d, the N 1s spectrum is resolved into two distinct characteristic peaks: the peak at 399.5 eV corresponds to unreacted primary amine (-NH_2_), while the peak near 401.6 eV belongs to secondary amine (-NH-). The appearance of the secondary amino signal indicates that the terminal amino groups of the aminated PDMS underwent a nucleophilic addition ring-opening reaction with the epoxy groups, thereby forming a crosslinked network and simultaneously improving the properties of the epoxy resin. The C-N/C-O bond component fitted at 285.8 eV in the C 1s spectrum (Figure 2e) indirectly confirms the formation of chemical bonds between the amino-terminated PDMS and the resin backbone [24,25].

As shown in the stress–strain curve in Figure 2f, the introduction of BaSO_4_ nanoparticles significantly enhanced the mechanical strength of the PDMS@EP matrix. Specifically, the tensile strength of the PDMS@EP-BaSO_4_ coating reached approximately 40.5 MPa, an increase of 68.8% compared with the 24.0 MPa of the control PDMS@EP sample. This significant enhancement was attributed to the rigid BaSO_4_ nanoparticles acting as effective cross-linking sites, promoting efficient load transfer within the polymer network and thereby improving the mechanical fracture strength of the coating.

### 3.2. Optical Properties of PDMS@EP-BaSO_4_ Coating

**Figure 3:**
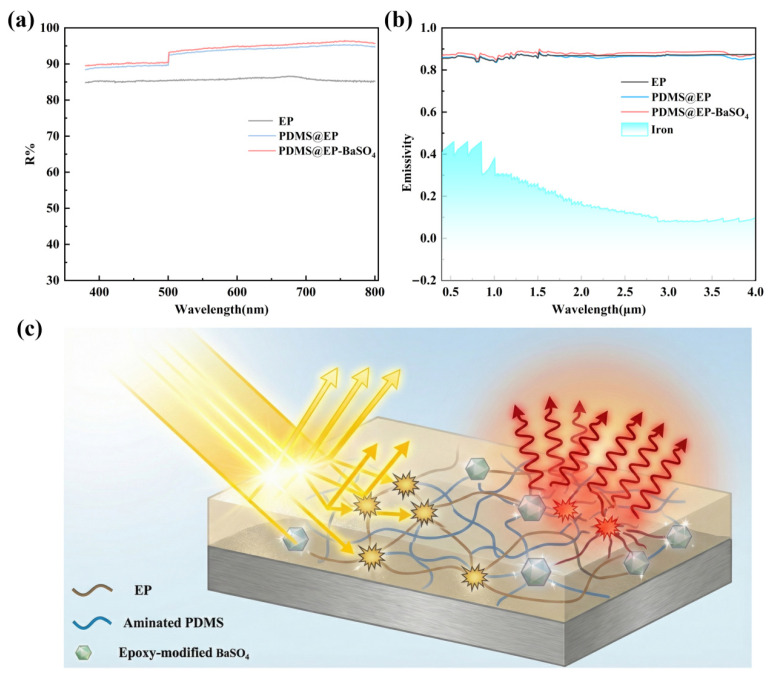
(**a**) Reflectance curves of EP, PDMS@EP and PDMS@EP-BaSO_4_ composite coatings; (**b**) emissivity curves; (**c**) schematic illustration of the structural regulation of solar reflectance and emissivity by the distribution of inorganic nanoparticles within the coating.

To characterize the optical properties of the composite coating, reflectance measurements were conducted in the visible light spectrum alongside emissivity testing within the atmospheric window. Figure 3a displays the spectral reflectance curves of the composite coating across the 380–780 nm range before and after modification. The PDMS@EP coating without added inorganic nanoparticles had a weighted average reflectance of about 91.5% in the visible light range. Following the incorporation of epoxy-modified BaSO_4_ nanoparticles, the reflectance curve of the composite coating exhibited a marked upward shift. The PDMS@EP- BaSO_4_ sample maintained a reflectance of approximately 95% within the visible spectrum, indicating that the modified particles and micro-nano composite structure within the matrix exerted a certain degree of light scattering. This indicated a partial refractive index mismatch between the high-refractive-index BaSO_4_ nanoparticles and the matrix. Such interfacial differences enhanced the Mie scattering efficiency within the coating, thereby boosting the light reflection of the substrate material. The emissivity spectrum in Figure 3b further corroborates the gain effect of the functionalized filler. Compared to the PDMS@EP coating, the composite coating incorporating modified barium sulphate exhibits an overall upward shift in spectral curves across the entire tested wavelength range, with markedly enhanced peak intensities approaching 90%. Moreover, the composite coating exhibits enhanced reflectance and emissivity compared to the pure EP coating.

These numerical changes demonstrate that introducing modified fillers superimposed additional radiative pathways onto the original coating properties, significantly boosting the material’s emissivity. Compared to the low emissivity of metals (~20%), this coating-enhanced approach substantially elevated metallic emissivity. Figure 3c illustrates the operating principle of the PDMS@EP- BaSO_4_ composite coating material, whose structure comprised an EP matrix embedded with an amino-functionalized PDMS network and epoxy-modified BaSO_4_ particles. This demonstrates how the coating reduced heat absorption through high solar reflectance and accelerated heat radiation via high infrared emissivity, thereby lowering metal surface temperatures without requiring additional cooling equipment. The high emissivity facilitates heat exchange through the cosmic atmospheric window, preventing metal heating under direct sunlight and offering broad prospects for applications in radiative cooling.

These numerical changes demonstrate that introducing modified fillers superimposed additional radiative pathways onto the original coating properties, significantly boosting the emissivity of the material. Compared to the low emissivity of metals (~20%), this coating-enhanced approach substantially elevated metallic emissivity. Figure 3c illustrates the operating principle of the PDMS@EP-BaSO_4_ composite coating material, whose structure comprised an EP matrix embedded with an amino-functionalized PDMS network and epoxy-modified BaSO_4_ particles. This demonstrated how the coating reduced heat absorption through high solar reflectance and accelerated heat radiation via high infrared emissivity, thereby lowering metal surface temperatures without requiring additional cooling equipment. The high emissivity facilitates heat exchange through the cosmic atmospheric window, preventing metal heating under direct sunlight and offering broad prospects for applications in radiative cooling.

### 3.3. Stability of PDMS@EP-BaSO_4_ Coating

**Figure 4:**
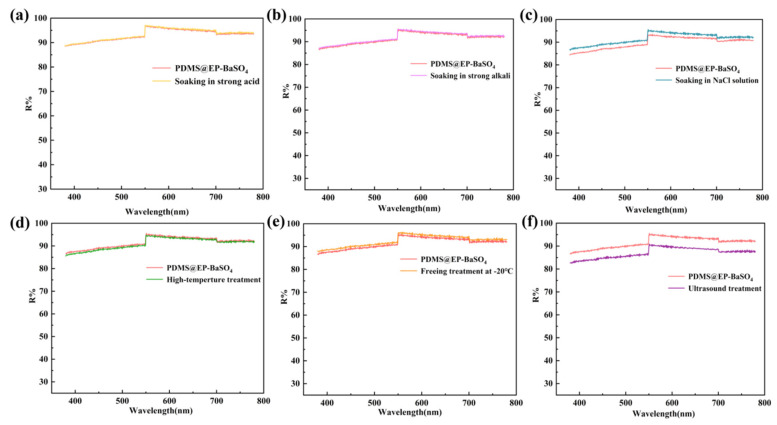
Environmental stability testing of the PDMS@EP-BaSO_4_ coating. Reflectance spectra of the coating before and after undergoing (**a**) immersion in 1 M HCl strong acid solution, (**b**) immersion in 1 M NaOH strong base solution, (**c**) immersion in 3.5 wt% NaCl salt solution, (**d**) storage at 150 °C (high temperature), (**e**) freezing at −20 °C (low temperature), and (**f**) 24 h ultrasonic treatment.

To verify the stability of the PDMS@EP-BaSO_4_ coating under harsh usage conditions, treatments were conducted for 24 h under different conditions, and the reflectance of the corresponding coating was tested. As shown in Figure 4, whether under strong acid, strong alkali, high-salt corrosive environments, or after 24 h of high-temperature heating and low-temperature freezing, the spectral reflectance of the coating in the 380–780 nm wavelength range remained highly consistent with that of the initial sample, with no significant decrease or spectral distortion observed. The results indicate that this coating possesses excellent weather resistance and stability. Additionally, after 24 h of intense ultrasonic shaking, the coating still maintained very high reflective performance, demonstrating that the modified BaSO_4_ nanoparticles and the epoxy–PDMS matrix form a strong interfacial bonding primarily through covalent bonds, supplemented by hydrogen bonds and van der Waals forces, rather than simple physical blending and curing. These strong forces endow the coating with excellent mechanical stability. In summary, the PDMS@EP-BaSO_4_ composite coating prepared through an open-ring addition reaction exhibits excellent environmental stability, can be applied in extreme high- and low-temperature conditions, and can maintain a high reflectance spectral enhancement performance to adapt to complex and variable application environments.

### 3.4. Wettability of PDMS@EP-BaSO_4_ Coating

**Figure 5:**
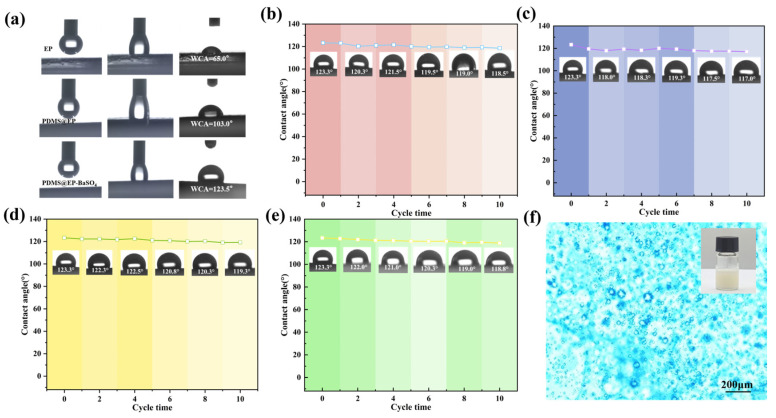
(**a**) Water contact angles of EP, PDMS@EP, and PDMS@EP-BaSO_4_ coatings. Water contact angle change curves of the PDMS@EP-BaSO_4_ composite coating after (**b**) sandpaper abrasion, (**c**) knife scraping, and (**d**) UV irradiation. (**e**) PDMS@EP-BaSO_4_ coating adhesion strength test. (**f**) Dispersion effect of the PDMS@EP-BaSO_4_ precursor emulsion under a microscope.

To evaluate the wetting properties of different composite coatings, water contact angle tests were conducted. As shown in Figure 5a, the original EP coating exhibited a WCA of 65°, consistent with the intrinsic hydrophilic wetting behavior of epoxy resin. The WCA of the modified PDMS@EP coating increased to 103°, a direct result of reduced surface energy due to PDMS incorporation. PDMS exhibited a surface energy of approximately 20 mJ/m^2^, significantly lower than the 45 mJ/m^2^ of EP. Their covalent bonding formed a crosslinked network, thereby decreasing the interfacial energy between the PDMS@EP coating and water. Following the incorporation of BaSO_4_ nanoparticles, the PDMS@EP-BaSO_4_ coating achieved a WCA of 123.5°. This enhancement primarily stems from the micro-nano scale roughness constructed by BaSO_4_ nanoparticles on the coating surface. Furthermore, this phenomenon aligns with the Cassie–Baxter model. The synergistic effect of the low surface energy of PDMS and the hierarchical roughness created by BaSO_4_ nanoparticles traps air cavities at the water–coating interface, significantly enhancing hydrophobicity [26].

To validate the durability of the composite coating, the PDMS@EP-BaSO_4_ coating underwent sandpaper abrasion, knife scraping, and UV irradiation tests. Changes in the water contact angle were recorded. Figure 5b shows that after 10 sandpaper abrasions, the water contact angle (WCA) decreased only slightly from 123.5° to 118.5°. This minimal change primarily stems from the robust structure formed by BaSO_4_ nanoparticles and epoxy resin, which effectively resists physical wear. Furthermore, Figure 5c indicates that even after 10 knife scratches, the contact angle remained around 117.0°. This demonstrates that the hydrophobic components were uniformly distributed within the coating. Even when the surface was scratched, the newly exposed areas retained their hydrophobicity. After UV irradiation cycles, Figure 5d shows that the WCA remained at 119.2° after 10 cycles, with no significant change. This stability primarily stems from the robust stability of Si-O bonds within PDMS molecular chains and the protective effect of BaSO_4_ on the coating [27]. These data indicate the coating maintains a stable performance under mechanical damage and outdoor light exposure. The results confirmed the PDMS@EP-BaSO_4_ coating exhibited outstanding chemical stability and resistance to UV-induced degradation.

As shown in Figure 5e, after repeated sticking and pulling of the adhesive tape, no residue appeared on the tape surface, the coating surface remained smooth, and the contact angle on the coating surface did not exhibit significant fluctuation, maintaining an average contact angle of 120°. Figure 5f displays a micrograph of the PDMS@EP-BaSO_4_ precursor emulsion in its emulsion state prior to curing, revealing a uniform emulsion phase.

### 3.5. Self-Cleaning Properties of PDMS@EP-BaSO_4_ Coating

**Figure 6:**
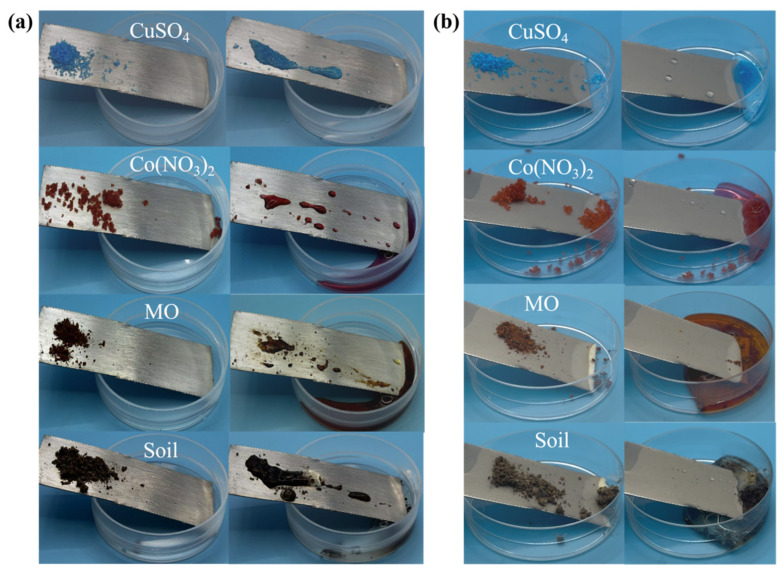
Photographs of water rinsing on surfaces with (**a**) metal plate; (**b**) PDMS@EP-BaSO_4_ composite coating.

To investigate the self-cleaning properties of the coating, in this study, we selected copper sulphate (inorganic salt), cobalt nitrate (metal salt), methyl orange (organic dye), and soil as representative simulated contaminants. The cleaning performance of untreated PDMS@EP-BaSO_4_ metal sheets was compared with that of coated metal sheets, as shown in Figure 6. The aforementioned contaminants were placed on both sample surfaces. After allowing 10 min for stable adhesion, the surfaces were rinsed for 30 s using deionized water simulating natural water flow at a velocity of 2 mL/s. Following the same rinsing procedure, the untreated blank metal plate (Figure 6a) retained substantial residues of all contaminants, cobalt nitrate showed the adherence of flaky deposits, methyl orange exhibited distinct droplet spreading with residual traces, and soil particles clung tenaciously. This indicated strong interfacial interactions between the bare metal substrate and contaminants, rendering simple water rinsing ineffective for thorough decontamination. In contrast, the PDMS@EP-BaSO_4_-coated metal plate exhibited physical retention of contaminants, including water-soluble salts (copper sulphate, cobalt nitrate), organic dye (methyl orange), and soil, on its surface without wetting or spreading (Figure 6b). Following deionized water rinsing, all contaminants were completely removed, restoring the sample to a pristine state. This phenomenon arises from the synergistic effect of the low surface energy and hierarchical rough structure of the PDMS@EP-BaSO_4_ coating. The low surface energy imparted by PDMS inhibits molecular adhesion between contaminants and the coating, while the hierarchical rough structure formed by nano-BaSO_4_ further reduces the effective contact area between contaminants and the coating. Consequently, the shear force of water flow sufficiently overcame interfacial adhesive forces, enabling efficient decontamination [28,29]. This integration of the coating with metallic substrates not only enhanced the reflectance and emissivity of the metal but also endowed the material with additional hydrophobic and self-cleaning properties.

### 3.6. Thermal and Stability Conductivity of PDMS@EP-BaSO_4_ Coating

**Figure 7:**
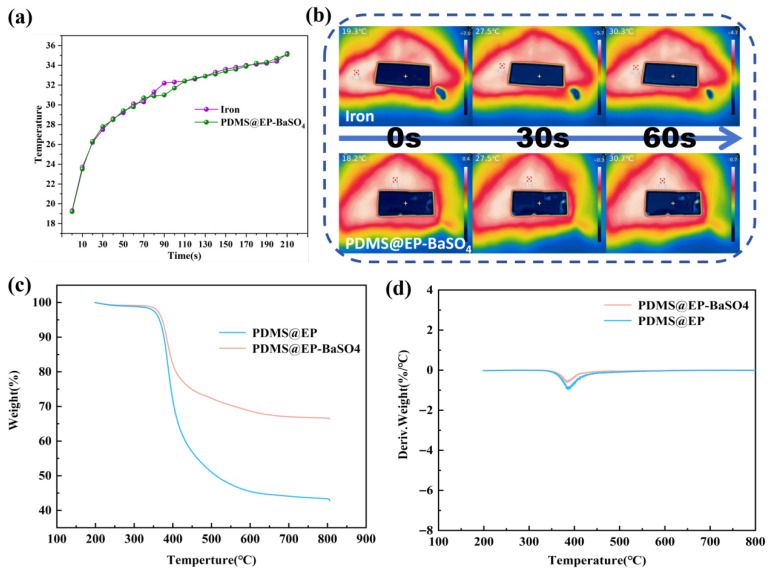
(**a**) Temperature change curves of the original metal sheet and the metal sheet coated with PDMS@EP-BaSO_4_; (**b**) infrared thermal imaging map. TG (**c**) and DTG (**d**) curves of PDMS@EP and PDMS@EP-BaSO_4_ coating.

Figure 7 illustrates the effect of the composite coating on the thermal conductivity of metallic materials. Metal specimens and samples coated with PDMS@EP-BaSO_4_ were placed on a 40 °C constant-temperature heating platform for testing. As shown in Figure 7a, with the increase in time, the metal sheet heated up from 19.3 °C on the surface to 35.2 °C, while the metal sheet coated with a PDMS@EP-BaSO_4_ coating heated up from 19.2 °C to 35.1 °C. The temperature difference between the uncoated metal sheet and the coated sheet remained consistently below 0.5 °C, indicating that the thermal resistance of the coating did not impair the thermal conductivity efficiency of the metal.

This was attributable to the coating matrix being prepared via composite formation of EP and amino-terminated PDMS. EP itself exhibited excellent thermal conductivity (approximately 0.2 W/(m·K)), while PDMS possessed a thermal conductivity of approximately 0.15 W/(m·K), placing it within the same order of magnitude as EP. No significant interfacial thermal resistance arose during the composite formation process, enabling uniform and continuous heat transfer within the matrix. Consequently, thermal resistance was lower compared to composite systems with significant thermal conductivity disparities between components. Crucially, the coating incorporated epoxy-modified BaSO_4_ nanoparticles particles, whose thermal conductivity (approximately 35 W/(m·K)) substantially exceeded that of the organic matrix. The uniform distribution of BaSO_4_ nanoparticles within the coating created thermal conduction pathways, further reducing thermal resistance and preventing impedance to heat transfer from the metal substrate. This implied that the composite coating applied to metal surfaces did not adversely affect the thermal conductivity or heating performance of the metal substrate [30,31]. The infrared heating map in Figure 7b visually demonstrates the thermal transfer efficiency at the coating–metal interface. At 0 s, both the PDMS@EP-BaSO_4_-coated metal sheet and the bare metal sheet exhibited identical initial temperatures. At 30 and 60 s, the core regions synchronously increased in temperature, indicating rapid heat conduction through the coating–metal interface. This phenomenon arose from the thin thickness of the coating (~300 μm) and the tight interface bonding between the coating and metal substrate. Heat could thus be rapidly transferred through the metal substrate, coating interface, and internal thermal pathways within the coating, meaning the coating did not substantially affect the thermal conductivity of the metal. In summary, this PDMS@EP-BaSO_4_ composite coating, prepared via an open-loop addition reaction incorporating BaSO_4_ inorganic nanoparticles, confers high reflectivity, high emissivity, and high thermal conductivity when applied to metallic surfaces. It offers significant application potential for high-performance developments in building energy efficiency, electronic heat dissipation, and solar energy utilization [32].

The thermal stability of the PDMS@EP coating and its composite coating PDMS@EP-BaSO_4_ was evaluated via thermogravimetric analysis (TG) and differential thermogravimetric analysis (DTG), with results presented in Figure 7c,d. Experimental findings indicate both coatings exhibited excellent thermal stability below 350 °C, with no significant mass loss observed. The primary degradation phase occurred between 350 and 550 °C, primarily attributable to thermal oxidative degradation of the EP matrix and chain scission of the PDMS segments.

Crucially, the PDMS@EP-BaSO_4_ composite coating exhibited a residual mass of 66.5% at 800 °C, substantially exceeding the 42.8% observed for the pristine PDMS@EP coating. This pronounced difference directly confirmed the effective loading of BaSO_4_ nanofillers within the coating matrix, which exhibit high thermal stability. Furthermore, as evident from the DTG curve in Figure 7d, although both samples exhibited maximum decomposition rate temperatures (Tmax) near 385 °C, the weight loss peak intensity was markedly reduced for the PDMS@EP-BaSO_4_ coating. This indicates that BaSO_4_ nanoparticles exert a pronounced physical barrier effect within the polymer matrix, effectively impeding heat transfer and retarding the volatilization of decomposition products [33]. In summary, the incorporation of BaSO_4_ nanoparticles not only enhanced the char yield through physical filling of the inorganic phase but also delayed the degradation process via a kinetic-level barrier effect, thereby substantially improving the stability of the composite coating under elevated temperatures.

## 4. Conclusions

This study proposed a strategy synergistically regulating open-ring addition reactions and micro and nano-structure construction, successfully preparing a multifunctional PDMS@EP-BaSO_4_ composite coating with high reflectance and high emissivity (90%). By chemically bonding low-surface-energy amino-terminated PDMS segments to the epoxy matrix and incorporating functionalized BaSO_4_ nanoparticles modified with dual silane coupling agents, it overcame the severe macroscopic phase separation and filler agglomeration issues inherent in conventional physical blending systems. The synergistic effect of this unique chemically induced wrinkling and hierarchical rough structure endowed the coating with exceptional hydrophobicity, significantly enhancing the water contact angle to 123.5° and demonstrating outstanding easy-to-clean performance against various complex contaminants. It also exhibited excellent mechanical strength, with tensile strength increased by 68.8% compared to coatings without BaSO_4_ nanoparticles.

Furthermore, the PDMS@EP-BaSO_4_ coating demonstrated exceptional environmental stability and mechanical robustness. After undergoing rigorous testing including strong acid–base corrosion, ultrasonic agitation, and extreme temperature cycling, neither its spectral enhancement properties nor interfacial adhesion exhibited significant degradation. This synergistic modification approach, combining long-term antifouling with high thermal conductivity efficiency, offers a novel technical pathway for the sustained protection of outdoor metal materials. Furthermore, the PDMS@EP-BaSO_4_ coating demonstrated exceptional environmental stability and mechanical robustness. After undergoing rigorous testing including strong acid–base corrosion, ultrasonic agitation, and extreme temperature cycling, neither its spectral enhancement properties nor interfacial adhesion exhibited significant degradation. This synergistic modification approach, combining long-term antifouling with high thermal conductivity efficiency, offers a novel technical pathway for the sustained protection of outdoor metal materials.

## Data Availability

The original contributions presented in this study are included in the article. Further inquiries can be directed to the corresponding author.
